# Examination of *Salmonella* Prevalence in Pigs Through Rye‐Based Feeding and Coarser Feed Structure Under Field Conditions

**DOI:** 10.1002/vms3.70041

**Published:** 2024-09-27

**Authors:** Jens Gerrit Lindhaus, Bernd Reckels, Bussarakam Chuppava, Richard Grone, Christian Visscher, Clara Berenike Hartung

**Affiliations:** ^1^ Institute for Animal Nutrition University of Veterinary Medicine Hannover, Foundation Hanover Germany; ^2^ KWS LOCHOW GmbH Bergen Germany

**Keywords:** field study, pig fattening, pig health, rye, *Salmonella*

## Abstract

**Introduction:**

Salmonellosis is the second most commonly occurring bacterial zoonosis in Germany. Rye in pig feeding offers new possibilities for addressing that issue due to its high content of non‐starch polysaccharides (NSPs). These are fermented in the intestinal tract to specific fermentation products, which seem to have bacteriolytic effects against *Salmonella*. A coarse feed structure can display synergistic effects.

**Methods:**

Seven conventional pig fattening farms increased the rye content (40%–70%) while limiting the amount of fine particles (maximum of 20% ≤0.25 mm). Samples from pigs were tested for *Salmonella* antibodies and compared with samples from 167 farms without any changes to the feed.

**Results:**

Rye‐based diets had a significant (*p* < 0.05) impact on *Salmonella* antibody (percentage optical density [OD%]) detection. In this study, it became apparent that significantly fewer positive OD% values could be detected due to the increase in rye compared to farms that did not change the feed (Farm 6 P0: 35.45 ± 36.18; P1: 15.48 ± 16.98; P2: 9.36 ± 8.17). An elimination of *Salmonella* could not be achieved, but especially farms with high antibody counts were able to strongly reduce those in both phases consecutively (Farm 5 P0: 35.17 ± 35.53; P1: 18.56^a^ ± 20.96; P2: 13.38^a^ ± 18.99). That was different on farms without adapted feeding, where an increase in *Salmonella* antibodies was observed (P0: 17.38 ± 22.21; P1: 20.12 ± 25.39; P2: 18.12 ± 25.44).

**Conclusion:**

By increasing the proportion of rye and limiting the proportion of fine particles in the feed, *Salmonella* antibodies (OD% values) in meat juice and blood can be significantly reduced, especially on farms with an initially high incidence of *Salmonella*. If that is implemented in feeding across the board on farms, an improvement in food safety and a decreased risk of zoonosis can be expected.

## Graphical abstract

In a field study in pig fattening, ‘experimental farms’ adapted their feeding to a higher proportion of rye and a coarser particle structure. At the slaughterhouse, blood and meat juice samples were tested for *Salmonella* antibodies and compared with samples from ‘control farms’ without adapted feeding. ‘Experimental farms’ could reduce the finding of *Salmonella* antibodies significantly compared to ‘control farms’ resulting in a reduced zoonosis risk in the production chain. *Source*: Image created with BioRender.com.

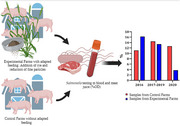

## 1 Introduction

Infections with *Salmonella*, especially the serovars *Salmonella Typhimurium* and *Salmonella* Enteritidis, are two of the most frequently associated bacterial zoonotic diseases in humans and are responsible for one of the largest shares of foodborne diseases worldwide (European Food Safety Authority [EFSA] 2023; Robert Koch‐Institut 2019). They are the second most commonly occurring bacterial foodborne disease in Germany (Robert Koch‐Institut 2021) and one of the most spread foodborne pathogens worldwide (Soliani et al. 2023). In pigs, the infections occur in all age groups and production types (Visscher et al. 2011), so that a monitoring system for systematic detection in Germany has become essential (Schweine‐Salmonellen‐Verordnung 2007). Thus, early intervention is indispensable to gain control over this issue (Kump and Löhren 2015).

**Summary**

- Changing the diet of pigs to a higher proportion of rye and increased/coarser structure can reduce *Salmonella* antibody counts in pigs.
- Especially on farms with high *Salmonella* antibody counts, rye and coarser feed structures can significantly reduce the finding of *Salmonella* antibodies, but no complete elimination can be achieved.
- Reduction of *Salmonella* occurrence appears to be possible even without feed additives, solely through adapted ingredients and structure of feed, which can help reduce the zoonotic risk caused by pork.

The most diffused feeding material in Europe for the composition of commercial diets for pigs accounts for cereals, commonly wheat and barley (Bundesanstalt für Landwirtschaft und Ernährung 2018). Rye (*Secale cereale*), mainly cultivated in northern Europe, is enjoying renewed attention (Persson et al. 2006). In 2022, 3159,410 ha of rye were harvested in Europe with a yield of 11,041,235.95 t (FAOSTAT 2024). Due to presumptions that non‐starch polysaccharides (NSPs) in rye could reduce feed intake and growth performance and concerns about ergot alkaloids, rye was rarely fed in the past (Friend and Macintyre 1970). However, recent progress in rye breeding has led to modern hybrids with not only a higher resistance against ergot contamination but have also been shown in studies to disprove lower feed consumption and performances (Jürgens, Jansen, and Wegener 2012; Miedaner and Geiger 2015; Wilke 2020). In recent years, studies have shown that a high proportion of rye with high dietary fibre content in the diet has positive effects in pig nutrition (Bach Knudsen 2019; Wilke 2020), and it was already shown that hybrid rye can replace wheat in the feeding of fattening pigs without disadvantages regarding performance (Chuppava et al. 2020). In addition, rye carbohydrates (NSP) are poorly digested in the small intestine, which means that a larger amount of fermentable substrate reaches the large intestine when rye is fed (McGhee and Hans 2018). A coarser feed composition additionally supports this process (McGhee and Stein 2020).

Rye contains the highest concentration of NSPs compared to other cereals (Rodehutscord et al. 2016; Wilke 2020). Especially arabinoxylans and fructans form a large proportion and are mainly fermented in the large intestine, which leads to the formation of short‐chain fatty acids (SCFAs) and especially butyrate in the cecum and colon (Bach Knudsen 2019; Glitsø et al. 1998). SCFA and especially butyrate have a bacteriostatic or bacteriolytic effect and have long been used in pig feeding to combat *Salmonella* (Visscher et al. 2009; Boyen et al. 2008; Van Immerseel et al. 2006). Butyrate is formed in the metabolism of anaerobic bacteria and supports intestinal health (Vital et al. 2017). This occurs inter alia because the butyrate interacts with the proteins of *Salmonella* and reduces the capacity of the pathogens to bind to the intestinal wall while also reducing penetration into the epithelial cells of the intestine. As an acid, butyrate lowers the pH value in the intestinal tract and changes the bacterial environment, which benefits immune response and intestinal health (Cappai et al. 2020). Butyrate even manages to interact with the *Salmonella* so that the invasion capability as well as the proliferation can be reduced (Hollmann et al. 2022). Consequently, a reduced *Salmonella* burden in pigs can be documented (Lawhon et al. 2002; Gantois et al. 2006). Thus, it is reasonable to conclude that a reduction in *Salmonella* load in animals would result from a change in the intestinal flora towards a butyrate‐rich fermentation pattern (Visscher et al. 2009; Boyen et al. 2008).

This field study therefore investigated whether a rye‐based diet (30%–70%) for fattening pigs can practicably and economically reduce the incidence of *Salmonella* detections at the abattoir due to its ingredients and effects in the intestine (NSPs, butyrate, pH value), in combination with a coarser feed structure and the associated positive influences on the intestinal tract.

## 2 Materials and Methods

The data collection was carried out in accordance with the Swine *Salmonella* Ordinance and in alignment with German law. The serological *Salmonella* condition took place during the slaughtering process, and no interventions were carried out on live animals as defined by the German Animal Welfare Act (animal welfare number: TVO‐2022‐V‐7). The aim of these investigations was to test feeding aspects for *Salmonella* serology in pig fattening. To conduct the study, only complete feed was used so that no lack of nutrients for the animals could occur. The course of the study was divided into three time phases: P0: 2016; P1: 2017–2019; P2: 2020. In addition, farms were divided into control farms (no change in feeding) and experimental farms (increased rye feeding, coarser feed structure: Farms 1–7). As can be seen in Table 1, all trial farms have implemented the specified feeding regime during the particular study period. All trial farms, as well as all control farms, belonged to the Aller‐Weser‐Hunte livestock marketing organization. The experimental farms have volunteered to take part in the study and adjust their feeding concept accordingly.

**TABLE 1:** Feed composition during the different phases for the experimental farms.

	Rye in %			Barley in %^a^	Size of particle mm (20% max. ≤)^a^	Lysine (g/MJ ME)^a^, ^b^
	Pre‐fattening	Mid‐fattening	End‐fattening			
Phase 1	5	20	40	25	0.25	0.75
Phase 2	>30	>40	45–70	15	0.25	0.75

^a^ Only at end‐fattening phase.

^b^ Minimum g lysine per MJ ME.

### 2.1 Farms

In total, data were available from 167 control farms and 7 experimental farms. All farms were located in the north‐west of Germany. The experimental farms ranged in size from 344 to 2000 fattening places. The type of livestock consisted almost exclusively of conventional husbandry (*n* = 6) with one exception of livestock farming in an outdoor climate with straw (*n* = 1). The farms had an average of three fattening runs per year.

Due to the fact that the data are based on a field study, the experimental farms could not be randomized further, as participation in the study was voluntary. Similarly, the data of the control farms were provided by livestock marketing and their catchment area, so that further information was not available to us.

### 2.2 Biosecurity of the Farms

For a better traceability of the serologic *Salmonella* situation, the experimental farms completed the Biosecurity Check Pig questionnaire from the University of Ghent, Belgium (Faculty of Veterinary Medicine). This check is a scientific scoring system to evaluate the quality of the internal and external biosecurity of their farms (Biocheck.Gent, Ghent University 2023), which means the better the biosecurity, the lower the risk of disease introduction (Biocheck.Gent, Ghent University 2023). A detailed version of the biosecurity check of the experimental farms is to be found in the supplementary data (Additional File 1).

### 2.3 Animals

The pigs on the control farms were conventionally housed pigs with uniform final slaughter weights bred under standardized procedures. A total of 50,253 pigs from the control farms were sampled in 3 phases.

A total of 3012 pigs from the experimental farms were sampled.

### 2.4 Feeding and Ration Design

#### 2.4.1 Control Farms

There was no further information provided concerning the feeding technologies of the control farms. The feeding design differed greatly and can be described as three experimental phases in the given years (P0 2016; P1 2017–2019; P2 2020).

#### 2.4.2 Experimental Farms

The farms (*n* = 7) used different types of feeding technology. The most common feeding technology was liquid feeding (*n* = 4). The other farms used feeding by means of sensor detection (*n* = 2) and pulp feeding (*n* = 1).

In Phase 0 (2016), there were no adjustments in the feeding concept. All experimental farms fed their different feeds with different compositions. No rye was included in the diets in that phase.

In Phase 1 (2017–2019), all farms had to adhere to a specific feeding schedule. A maximum of 20% of the particles in the ground feed or mixtures were allowed to have a size of ≤0.25 mm. The proportion of rye in the pre‐fattening phase (body mass: 28–60 kg) had to be 5%, in the middle fattening phase (body mass: 60–80 kg) 20% and in the final fattening phase (body mass: >80 kg) 40%. In addition, the feed mixture in the finishing phase had to contain 25% barley and ensure a minimum ratio of 0.75 g lysine to energy (1 MJ ME, see Table 1).

In Phase 2 (2020) of the study, the following guideline values were set for the newly defined feeding concept: The proportion of rye in the pre‐fattening phase had to be ≥30%, in the mid‐fattening phase ≥40% and in the finishing phase 60%–70%. The barley proportion in the final fattening phase had to be 15% of the compound feed. The particle sizes of the ground feed or mixtures (20% maximum ≤0.25 mm) and the lysine content (minimum 0.75 g lysine/MJ ME in the final fattening phase) were the same as in Phase 1 (2017–2019) of the studies. An overview of the different phases can be found in Table 1.

### 2.5 Data Collection/*Salmonella* Testing

A total of 53,265 individual samples from 174 farms were compared concerning the serologic *Salmonella* situation, time periods, sample type and feeding concept (167 control farms with 50,253 samples; seven experimental farms with 3012 samples).

The pigs that were fattened under the specified parameters were transported to different slaughterhouses in the north‐west of Germany after reaching slaughter weight. All the steps described below for obtaining the data refer to the quality assurance methods (Leitfaden QS Salmonellenmonitoring 2023). In a standardized procedure (Leitfaden QS Salmonellenmonitoring 2023), blood and meat juice samples were obtained. For this purpose, pieces of the diaphragm pillar were taken for the meat juice sample during the slaughtering process. The meat juice could be produced by comminuting the muscle parts.

The blood samples were collected after the pig had been stabbed in the process of slaughter. The samples could only be used if they had not been taken earlier than 14 days before slaughter. They served to complete the data and were not allowed to replace the collection of samples at the slaughterhouse. After centrifugation in the laboratory, the blood plasma/serum could be used for further investigations.

The detection of *Salmonella* antibodies in the blood and meat juice samples was determined by means of state‐approved ELISA test kits (Herd Check; PrioCheck *Salmonella* 2.0; pigtype *Salmonella* Ab), which were used to detect antibodies of the cell wall components of *Salmonella*. For this purpose, a drop of the blood plasma/serum or meat juice was placed onto the test kit. To ensure comparability of the results from the different ELISA test kits, the results were converted into a percentage optical density value (OD%). This percentage value has a conversion factor and was related to the positive and negative controls. A positive *Salmonella* result, according to the Swine *Salmonella* Ordinance, was said to exist if the OD% value was at least 40. All pigs that had an OD value greater than 40% were designated as *Salmonella* positive. In addition, in this study, we examined a cut‐off value of 10% as positive in order to understand how low the *Salmonella* prevalence can decrease. Each individual result was included in the statistical analysis. This serological test on carcasses is defined by the Pig *Salmonella* Regulation of Germany and is only carried out in Europe.

### 2.6 Statistical Analysis

Data were recorded in Microsoft Excel 2016 (Microsoft Corp., Redmond, WA, USA) and statistically analysed using SAS v. 7.1 (SAS Inst. Inc. Cary., NC, USA). Data were examined for their normal distribution using the Kolmogorov–Smirnov and Shapiro–Wilk tests. For group comparison for normally distributed data, the *t* test was applied, and for non‐normal data, the unpaired two‐samples Wilcoxon test. The differences between the individual phases within a farm as well as between the farms within a phase were analysed using the described tests. The OD values of individual pigs on the farms were compared with each other; an OD greater than or equal to 40 describes a positive pig. The significance level was specified with *p* < 0.05.

## 3 Results

### 3.1 *Salmonella* Serology in Percentage With Increased Rye Feeding and Non‐Specific Feeding Over the Years

**FIGURE 1:**
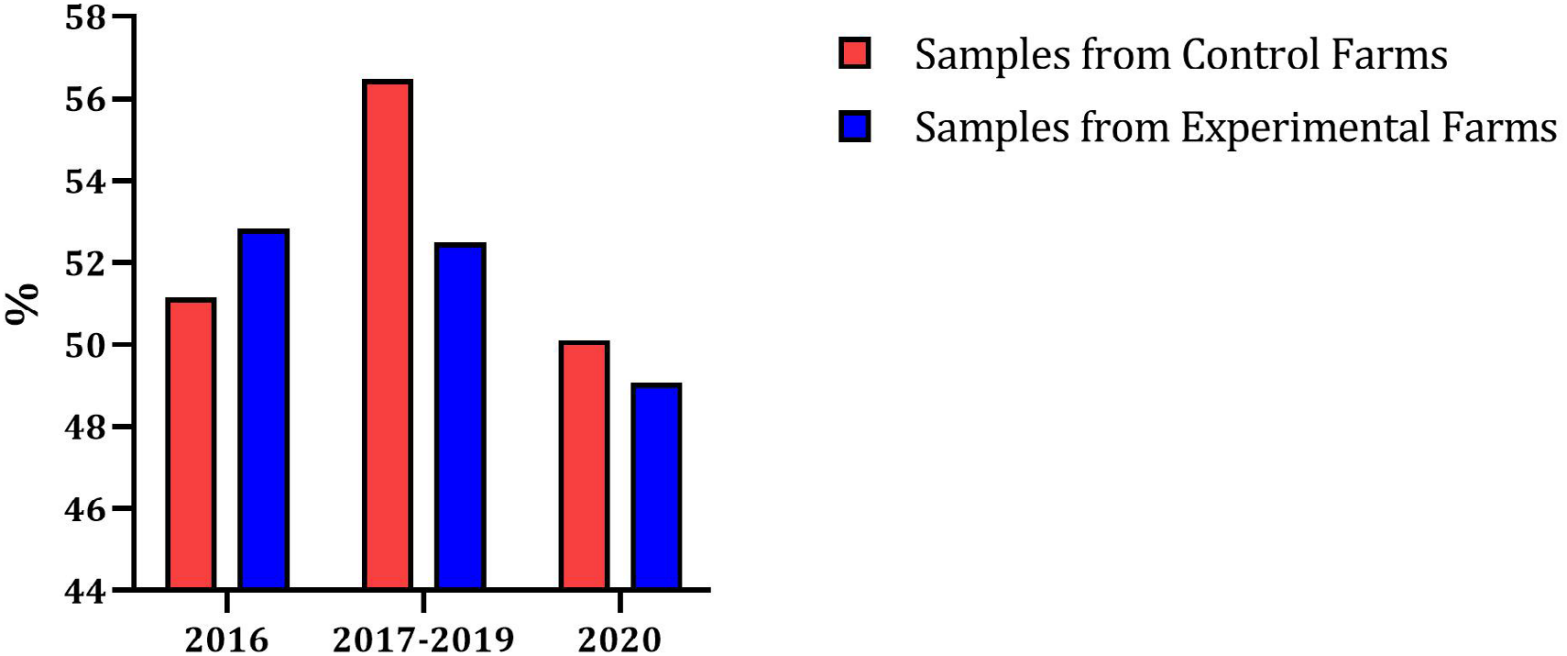
Percentage of positively tested animals (OD% value ≥10) in three different feeding phases on farms with and without rye feeding. OD%, percentage optical density.

Figure 1 shows the percentage of pigs with an OD% value of ≥10 differentiated into whether the samples stem from control farms (red) or experimental farms (blue) and in the three different feeding phases (P0: 2016; P1: 2017–2019; P2: 2020).

In 2016 (P0), the control farms start with a value of 51.15% and the experimental farms with 52.82%. In the years 2017–2019 (P1), the control farms increase to 56.49% and the experimental farms decrease to 52.49%. In the last year of the 2020 field study (P2), the *Salmonella* prevalence on both farm types decreases. The control farms stand at 50.11% and the experimental farms at 49.07%. All values refer to an OD% value of ≥10.

**FIGURE 2:**
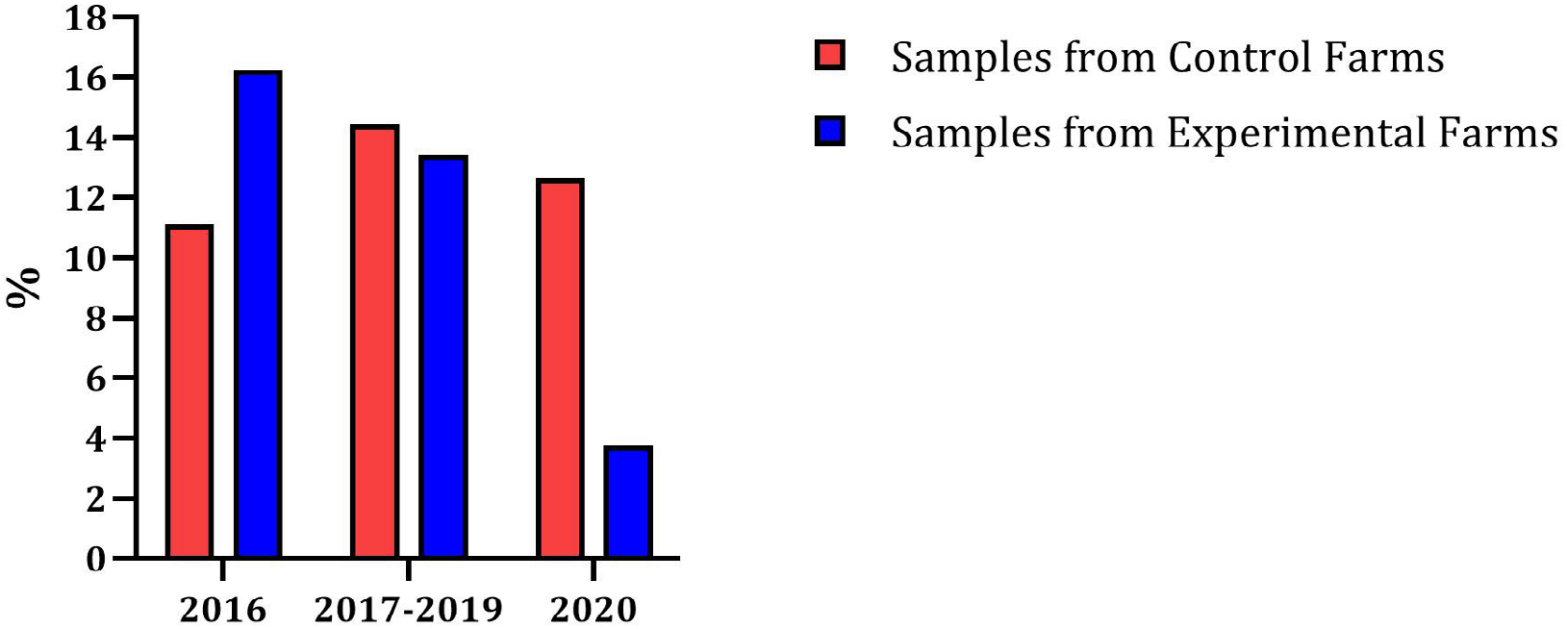
Percentage of positively tested animals (OD% value ≥40) in three different feeding phases on farms with and without rye feeding. OD%, percentage optical density.

Figure 2 shows the OD% of all pigs tested positive (OD ≥ 40) differentiated into whether the samples stem from control farms (red) or experimental farms (blue) and in the three different feeding phases (P0: 2016; P1: 2017–2019; P2: 2020).

In 2016 (P0), the control farms start with 11.12% and the experimental farms with 16.24%. In the following years 2017–2019 (P1), the control farms increase to 14.43% and the experimental farms decrease to 13.43%. In the last study year 2020 (P2), the control farms dropped to 12.65% and the experimental farms dropped significantly to 3.75%. All percentages refer to an OD% value of ≥ 40.

### 3.2 *Salmonella* Serology for Farms, Sample Type and Feeding Phase

Table 2 shows the mean values and standard deviations of the OD values divided by farms (control farms/experimental farms), number of sampled pigs with differentiation by type of sampling (meat juice/blood) and feeding period (P0 [2016]/P1 [2017–2019]/P2 [2020]).

**TABLE 2:** OD values (%) for 174 farms (167 control farms and 7 experimental farms) in 3 different feeding phases (blood vs. meat juice, mean ± SD).

Phase	Type of sample	Control farms		Experimental farms	
		*n*	OD value	*n*	OD value
P0	Blood	3423	16.01^[Link]^, ^[Link]^ ± 20.45	105	19.37^aC^ ± 22.96
	Meat juice	7307	18.02^[Link]^, ^[Link]^ ± 22.96	445	21.05^bC^ ± 29.12
P1	Blood	13,067	18.37^bC^ ± 23.01	796	15.83^[Link]^, ^[Link]^ ± 17.27
	Meat juice	17,897	21.39^[Link]^, ^[Link]^ ± 26.93	1148	15.99^[Link]^, ^[Link]^ ± 18.45
P2	Blood	3472	17.26^[Link]^, ^[Link]^ ± 24.87	298	12.51^[Link]^, ^[Link]^ ± 10.41
	Meat juice	5088	18.70^[Link]^, ^[Link]^ ± 25.80	220	11.63^[Link]^, ^[Link]^ ± 13.31

Abbreviation: OD%, percentage optical density.

^a,b^Averages differ significantly within a row (*p *< 0.05).

^A,B,C^Averages differ significantly within a column (*p* < 0.05) (comparison exclusively within the same sample type).

Concerning the OD values differentiated by farms, several significant differences can be observed. At the beginning of the investigations in Phase 0 (2016), the control farms started with significantly lower OD values in the meat juice samples (18.02 ± 22.96) compared to the experimental farms (21.05 ± 29.12). In the course of time, this proportionality changed, and already in Phase 1 (2017–2019), the experimental farms showed significantly lower OD values (15.99 ± 18.45) than the control farms (21.39 ± 26.93). The same occurred in Phase 2 (2020), where the experimental farms continued to show significantly lower OD values in the meat juice samples (11.63 ± 13.31) than the control farms (18.70 ± 25.80). When looking at the meat juice samples in the individual phases for the control farms only or for the experimental farms, it is evident for the control farms that there was a significant increase in the OD values from Phase 0 (2016: 18.02 ± 22.96) to Phase 1 (2017–2019: 21.39 ± 26.93), which then decreased significantly in Phase 2 (2020: 18.70 ± 25.80). The OD values of the meat juice samples from the experimental farms decreased significantly in the course of the three feeding phases (P0 2016: 21.05 ± 29.12; P1 2017–2019: 15.99 ± 18.45; P2 2020: 11.63 ± 13.31). For the blood samples, significant differences could be found in Phase 1 (2017–2019), where the experimental farms (15.83 ± 17.27) showed significantly lower OD values than the control farms (18.37 ± 23.01). These significantly lower values could also be reproduced by the experimental farms (12.51 ± 10.41) in Phase 2 (2020) for the blood samples in comparison to the control farms (17.26 ± 24.87). Focusing on the farms alone, the OD values of the blood samples for the control farms showed clear fluctuations in the three phases. From Phase 0 (2016: 16.01 ± 20.45) to Phase 1 (2017–2019: 18.37 ± 23.01), the values increased significantly, only to decrease again significantly in Phase 2 (2020: 17.26 ± 24.87). For the experimental farms, a progression of OD values that steadily decreased could be seen. At the start in Phase 0 (2016: 19.37 ± 22.96), the experimental farms showed the highest OD values, which then decreased significantly in Phase 1 (2017–2019: 15.83 ± 17.27) and from Phase 1 (2017–2019) to Phase 2 (2020: 12.51 ± 10.41).

Table 3 shows the OD means and standard deviations of the experimental farms compared to the control farms in total with the number of pigs (*n*) in three feeding phases (P0 [2016]/P1 [2017–2019]/P2 [2020]) without differentiation by sample type.

**TABLE 3:** OD values (%) for seven experimental farms and control farms in three different feeding phases (P0/P1/P2) without differentiation into blood and meat juice (mean ± SD).

	P0		P1		P2	
Farms	*n*	OD value	*n*	OD value	*n*	OD value
1	77	10.66^a^ ± 12.87	222	20.17^b^ ± 22.51	72	10.24^a^ ± 10.33
2	72	10.19^a^ ± 13.75	238	14.84^a^ ± 17.14	75	12.07^a^ ± 8.42
3	68	12.94^a^ ± 14.38	255	14.75^a^ ± 13.51	68	12.09^a^ ± 8.91
4	58	10.20^a^ ± 13.58	260	17.83^b^ ± 16.77	83	15.24^b^ ± 12.62
5	118	35.17^b^ ± 35.53	399	18.56^a^ ± 20.96	66	13.38^a^ ± 18.99
6	107	35.45^c^ ± 36.18	358	15.48^b^ ± 16.98	68	9.36^a^ ± 8.17
7	50	9.51^ab^ ± 16.65	212	7.63^a^ ± 11.08	86	12.09^b^ ± 11.03
Control	10,730	17.38^a^ ± 22.21	30,964	20.12^b^ ± 25.39	8560	18.12^ab^ ± 25.44

Abbreviation: ND, no data; OD%, percentage optical density.

^a,b,c^Averages differ significantly within a row (*p* < 0.05).

Significant differences could already be seen on Farm 1, with an increase in OD values from Phase 0 (2016: 10.66 ± 12.87) to Phase 1 (2017–2019: 20.17 ± 22.51) and a decrease in Phase 2 (2020: 10.24 ± 10.33). For Farm 4, there was a significant increase in OD values in Phase 1 (2017–2019: 17.83 ± 16.77) and Phase 2 (2020: 15.24 ± 12.62) compared to Phase 0 (2016: 10.20 ± 13.58). The first decreases in OD values could be seen on Farm 5, with a significant difference from Phase 0 (2016: 35.17 ± 35.53) to Phase 1 (2017–2019: 18.56 ± 20.96). In Phase 2 (2020: 13.38 ± 18.99), the values remained at a low level. Farm 6 showed the greatest decrease, which was significant throughout all three phases. With a high starting value in Phase 0 (2016: 35.45 ± 36.18), OD values decreased in Phase 1 (2017–2019: 15.48 ± 16.98) and again in Phase 2 (2020: 9.36 ± 8.17). Farm 7 displayed the lowest OD value in Phase 0 (2016: 9.51 ± 16.65). In Phase 1 (2017–2019: 7.63 ± 11.08) and Phase 2 (2020: 12.09 ± 11.03), so the values remained at a constantly low level. The control farms started with a medium‐to‐high OD value in Phase 0 (2016: 17.38 ± 22.21) and increased the values to the following phase (2017–2019: 20.12 ± 25.39), where there was a significant difference. In the last phase (2020: 18.12 ± 25.44), the level was maintained without significant changes.

Table 4 shows the means and standard deviations of the OD values of the individual farms and the control farms, the number of sampled pigs (*n*) and the feeding phase by period (P0 [2016]/P1 [2017–2019]/P2 [2020]). This table refers exclusively to the data obtained by blood sampling.

**TABLE 4:** OD values (%) for seven experimental farms and control farms in three different feeding phases, specific to the farm and sample (blood, mean ± SD).

	P0		P1		P2	
Farms	*n*	Blood	*n*	Blood	*n*	Blood
1	17	19.27^ab^ ± 12.24	160	22.69^b^ ± 24.91	72	10.24^a^ ± 10.33
2	18	14.40^a^ ± 20.18	213	14.40^a^ ± 16.85	75	12.07^a^ ± 8.42
3	14	16.99^a^ ± 12.07	194	13.50^a^ ± 11.16	68	12.09^a^ ± 8.91
4	5	13.74^a^ ± 6.76	174	16.16^a^ ± 15.22	83	15.24^a^ ± 6.18
5	33	18.96^a^ ± 21.18	14	10.85^a^ ± 7.32	ND	ND
6	18	28.60^a^ ± 36.39	19	9.79^b^ ± 7.45	ND	ND
7	ND	ND	22	6.31 ± 3.80	ND	ND
Control	3423	16.01^a^ ± 20.45	13,067	18.37^b^ ± 23.01	3472	17.26^ab^ ± 24.87

Abbreviation: ND, no data; OD%, percentage optical density.

^a,b^Averages differ significantly within a row (*p* < 0.05).

Comparing the blood samples from Farm 1 in phases P1 (2017–2019) and P2 (2020), a decrease in OD values with a significant difference became evident. Farms 2–4 displayed slight decreases and increases, but without any significance. No blood samples could be obtained on Farms 5 and 6 in Phase 2 (2020). Comparing Phase 0 (2016) with Phase 1 (2017–2019), Farm 6 displayed a significant decrease in values from 28.60 ± 36.39 to 9.79 ± 7.45. On Farm 7, blood samples were only collected in Phase 1 (2017–2019), so a comparison was not possible. The samples tested showed low OD values (6.31 ± 3.80). On the control farms, OD values increased from Phase 0 (2016: 16.01 ± 20.45) to Phase 1 (2017–2019: 18.37 ± 23.01) and represented a significant difference.

Table 5 shows the means and standard deviations of the OD values of the experimental farms and the control farms, the number of sampled pigs (*n*) and the feeding phase by period (P0 [2016]/P1 [2017–2019]/P2 [2020]). This table refers exclusively to the data of the meat juice samples.

**TABLE 5:** OD values (%) for seven experimental farms and control farms in three different feeding phases specific to the farm and sample (meat juice, mean ± SD).

	P0		P1		P2	
Farm	*n*	Meat juice	*n*	Meat juice	*n*	Meat juice
1	60	8.22^a^ ± 12.05	62	13.66^b^ ± 12.62	ND	ND
2	54	8.48^a^ ± 10.71	25	18.57^b^ ± 19.48	ND	ND
3	54	11.08^a^ ± 14.77	61	18.72^b^ ± 18.75	ND	ND
4	53	9.87^a^ ± 14.05	86	21.20^b^ ± 19.20	ND	ND
5	85	41.47^a^ ± 37.32	385	18.84^b^ ± 21.24	66	13.38^b^ ± 18.99
6	89	36.84^a^ ± 36.19	339	15.80^b^ ± 17.31	68	9.36^c^ ± 8.17
7	50	9.51^a^ ± 16.65	190	7.78^a^ ± 11.63	86	12.09^a^ ± 11.03
Control	7307	18.02^a^ ± 22.96	17,897	21.39^b^ ± 26.93	5088	18.70^a^ ± 25.80

Abbreviation: ND, no data; OD%, percentage optical density.

^a,b^Averages differ significantly within a row (*p* < 0.05).

On Farms 1–4, no meat juice samples could be obtained in Phase 2 (2020). On comparing the low OD values of the farms from Phase 0 (2016) with Phase 1 (2017–2019) on the individual farms, respectively, there was an increase on all farms with a significant difference. Moreover, Farm 5 showed a significant difference, with high OD values in Phase 0 (2016: 41.47 ± 37.32), the values decreased in Phase 1 (2017–2019: 18.84 ± 21.24). In Phase 2 (2020: 13.38 ± 18.99), no further changes were displayed. Farm 6 started with high OD values (36.84 ± 36.19) in Phase 0 (2016). Both in subsequent Phase 1 (2017–2019: 15.80 ± 17.31) and Phase 2 (2020: 9.36 ± 8.17), the values could be significantly reduced. On Farm 7, no significant differences were visible; the OD values were at a constantly low level. On the control farms, there was an increase from Phase 0 (2016: 18.02 ± 22.96) to Phase 1 (2017–2019: 21.39 ± 26.93) with a significant difference. In Phase 2 (2020: 18.70 ± 25.80), the OD values also decreased significantly to the same level as in Phase 0 (2016).

## 4 Discussion

*Salmonella*‐associated gastrointestinal infections in humans continue to be a high concern in Germany, Europe and worldwide (European Food Safety Authority 2023; Robert Koch‐Institut 2019, 2021). The European Union has set targets to combat *Salmonella* and other foodborne diseases (EC No. 2160/2003). This study investigated the relationship between rye‐based and coarse structured feeding and its effect on *Salmonella* OD% values found at the slaughterhouse in fattening pigs under field conditions.

Initial success of the tested feeding concept can already be seen in Figures 1 and 2. Figures created as an overview and progression of the pigs tested positive (OD% value ≥40/≥10) on control and experimental farms show a reduction in *Salmonella*‐positive pigs on the experimental farms over the course of the study. Particularly in Figure 2, in which the pigs were evaluated as positive from an OD% value of ≥40, the positive *Salmonella* testings were reduced from 16.24% (P0) to 3.75% (P2). But even in Figure 1, where the limit value is already ≥10%, which is lower than in the ‘Swine *Salmonella* Regulation’ in Germany (Schweine‐Salmonellen‐Verordnung 2007), the positive pigs are reduced from 52.82% (P0) to 49.07% (P2). This means that *Salmonella* prevalence decreases with rye‐based and coarser feed. This assumption is supported by the control farms. These farms also reduce their *Salmonella* prevalence in P2 (Figure 1: 50.11%; Figure 2: 12.65%) but are subject to significant deviations over the course of the study (P0–P2).

The results can be linked to the descriptions of Kamphues et al. (2007), who, in the absence of rye addition, successfully reduced *Salmonella* counts in the cecal content using a coarser feed structure. In that study, a coarser feed structure (62.2% of particles >1 mm; 10% <0.4 mm) and the addition of acids (1.2% potassium diformate) in piglets resulted in reduced *Salmonella* prevalence (Kamphues et al. 2007). Increasing particle size in the diet increases starch influx into the cecum and colon and therefore increases the propionic and butyric acid concentration in the chyme, which is thought to reduce *Salmonella* invasion (Boyen et al. 2008; Van Immerseel et al. 2006; Tan et al. 2021). In the present study, it could be shown that even without the use of acids, a lower *Salmonella* antibody count was observable.

The results in Table 2 confirm these statements. With a significance of *p* < 0.05, the *Salmonella* prevalence decreases with an increase in the rye content (P0: 19.37 ± 22.96; P1: 15.83 ± 17.27; P2: 12.51 ± 10.41). A reduction was observed in both the meat juice and blood samples on the experimental farms. Thus, a positive statistical influence of a coarser feed structure and a higher proportion of rye can also be determined. In addition, rye is usually more affordable than wheat and supports the farmer financially. To our knowledge, using high amounts of hybrid rye has been previously studied to control *Salmonella* in young pigs (Chuppava et al. 2020). The authors reported that young pigs experimentally infected with *S*. *Typhimurium* had significantly lower *Salmonella* counts in the cecum chyme after being fed a diet containing 69% rye compared to a diet containing 69% wheat with otherwise the same ingredients and the same formulation (Chuppava et al. 2020).

Fabà et al. (2020) reported that pigs fed an experimental diet (barley 23.3%, wheat 20%, corn 18%, wheat bran 3%, soybean meal 17.3%, potato protein 2.25%) with added organic acids (0.4%) combined with fermented rye (0.2%) had reduced *S*. *Typhimurium* shedding compared to animals fed added organic acids plus coated butyrate (3.11 and 3.87 log10 CFU/g, respectively) over the 21‐day period post‐challenge with 10^9^ CFU/mL. Although the studies cannot fully be equated with each other, this study shows that a reduction in *Salmonella* prevalence can also be achieved in pigs on farms by solely feeding distinct diets, which means that increasing the rye content can minimize *Salmonella* antibody findings even without adding organic acids, which means that rye improves intestinal health thus reducing veterinary costs and makes the addition of acids superfluous, which reduces costs even more.

When considering the farms individually in Table 3, we gain new insights. Farms with a high *Salmonella* burden, such as Farm 6 (P0: 35.45 ± 36.18), were able to significantly (*p* < 0.05) reduce their levels after feeding the diet, and a reduction took place after embedding 40% rye into the diet (P1: 15.48 ± 16.98) as well as with up to 70% of rye (P2: 9.36 ± 8.17). Farms with already low infection rates, however, did not show significant changes. Farm 7 showed consistently low infection numbers in the three feeding phases (P0 2016: 9.51^ab^ ± 16.65; P1 2017–2019: 7.63^a^ ± 11.08; P2 2020: 12.09^b^ ± 11.03). A further reduction in prevalence of *Salmonella* antibodies could not be achieved. Prevalence was at a consistently low level and could be kept low by rye‐based feeding. Increasing the proportion of rye up to 70% also did not lead to a further reduction in antibody prevalence. In a previous study by Sander et al. (2012), 60 weaner piglets were fed coarser and fine feed particles and then tested for their bacterial counts in the gastrointestinal tract. The coarser particle group was found to have significantly higher Lactobacilli counts and a pronounced pH gradient in the stomach contents (cardia, 5.15 ± 0.475; pylorus, 2.83 ± 1.06; *p* < 0.01), resulting in the lowest numbers of coliform bacteria at the distal end of the small intestine (Sander 2012). Those findings may explain the outcome displayed in Table 3 even though a direct transfer of the results to our study is not possible. An improvement in the intestinal flora such as found by Hankel et al. (2022), especially in the distal section of the small intestine, may have made it more difficult for *Salmonella* to bind. OD% values decreased over the course of the feeding phases. It remains unclear why those farms with low *Salmonella* counts could not further improve their situation. Furthermore, it is yet to be seen whether the coarser structure and different components of rye alter the intestinal flora of pigs to create the worst possible environment for *Salmonella* but fail to completely eliminate the presence of *Salmonella*. This would require further investigation.

On the basis of the results obtained, no differences in the prevalence of *Salmonella* antibodies among the sample types could be described (Tables 4 and 5). This means that we can rule out contamination, which is mainly found in meat juice samples from blood at the slaughterhouse. In fact, we can confirm the previous results. Low *Salmonella* prevalences in P0 could not be further reduced (Table 4: Farm 2: P0 2016: 14.40 ± 20.18 P1 2017–2019: 14.40 ± 16.85 P2 2020: 12.07^a^ ± 8.42); high *Salmonella* prevalences are reduced by the use of rye‐based feeding (Table 5: Farm 6: P0 36.84 ± 36.19 P1 15.80 ± 17.31 P2 9.36 ± 8.17).

Consideration of using blood serum or meat juice for the diagnosis of *Salmonella* infection in pigs has been reported in previous studies. Viana et al. (2020) suggested that meat juice can be considered an alternative to blood serum as a matrix for ELISA for preliminary detection of *Salmonella*, allowing the identification of potential sources of contamination during slaughtering. In contrast, Visscher et al. (2009) demonstrated that safe sampling is particularly important, as meat juice samples contaminated with blood showed higher levels of *Salmonella* antibodies than non‐contaminated samples. This could be attributed to higher antibody counts in the blood (Visscher et al. 2009). That could also explain the results in Table 5 of Farm 1, where an increase in OD% value from P0 (2016) to P1 (2017–2019) can be seen.

On farms with a high prevalence of *Salmonella* antibodies, the amount of antibodies could be reduced with an increase in the proportion of rye. On farms with low infection levels, rye may not cause any further reduction.

Our study shows that farms with known *Salmonella* problems can significantly reduce their *Salmonella* serology by changing the grain variety to rye and to a coarser feed structure. *Salmonella* could be reduced both at levels of 40% and up to 70%. A reduction at already low *Salmonella* levels, on the other hand, was not observed. In addition, in contrast to the studies by Wilke (2020), an older study found a reduction in weight gain in pigs when more than 50% of the barley and wheat content was replaced with rye. However, our study supports findings of improved gut health and increased levels of butyrate from rye NSPs (Bach Knudsen et al. 2005). This in turn can reduce the growth of *Salmonella* (Hollmann et al. 2022).

## 5 Conclusion

In conclusion, it remains to be said that the occurrence of *Salmonella* in the slaughterhouse and also in the production chain is still widespread. However, we can reduce the prevalence of *Salmonella* by adapting the feed on the farm to a rye‐based, coarser feed. Especially on farms with high *Salmonella* burden, *Salmonella* prevalence can be significantly reduced within few years. Accordingly, it is recommended that farms with high *Salmonella* prevalence in particular switch to a rye‐based, coarse ground feed. This can help to reduce the spread of *Salmonella* in the production chain and environment.

## Author Contributions

Conceptualization, funding acquisition, methodology and resources: Richard Grone, Clara Berenike Hartung and Christian Visscher. Data curation and formal analysis: Clara Berenike Hartung, Jens Gerrit Lindhaus and Bernd Reckels. Investigation: Richard Grone, Jens Gerrit Lindhaus and Bernd Reckels. Project administration and supervision: Clara Berenike Hartung and Christian Visscher. Software: n.a. Validation: Clara Berenike Hartung, Jens Gerrit Lindhaus and Christian Visscher. Visualization: Bussarakam Chuppava, Clara Berenike Hartung, Jens Gerrit Lindhaus and Christian Visscher. Writing–original draft: Bussarakam Chuppava, Clara Berenike Hartung, Jens Gerrit Lindhaus and Bernd Reckels. Writing–review and editing: all authors.

## Ethics Statement

Animal experiments were carried out in accordance with German regulations. Because no relevant interventions according to the Animal Protection Act (§ 7, Paragraph 2, Sentence 3) had been carried out on live animals, the study was not an animal experiment, and thus did not require approval from the competent authority (The serologic testing took place during the slaughtering process and no interventions were carried out on live animals—animal welfare number: TVO‐2022‐V‐7).

## Conflicts of Interest

The authors declare no conflicts of interest.

## Supporting information



Supporting Information

## Data Availability

The datasets used and analysed during the current study are available from the corresponding author on reasonable request.

## References

[vms370041-bib-0003] Bach Knudsen, K. E. 2019. “Nutritional Modulation to Improve Health and Welfare.” In Poultry and Pig Nutrition: Challenges of the 21st Century, edited by W. H. Hendriks , M. V. A. Verstegen , and L. Babinszky , 87–96. Wageningen, The Netherlands: Wageningen Academic Publishers.

[vms370041-bib-0004] Bach Knudsen, K. E. , A. Serena , A. K. Bjørnbak Kjær , H. Jørgensen , and R. Engberg . 2005. “Rye Bread Enhances the Production and Plasma Concentration of Butyrate but Not the Plasma Concentrations of Glucose and Insulin in Pigs.” Journal of Nutrition 135, no. 7: 1696–1704. 10.1093/jn/135.7.1696. 15987852

[vms370041-bib-0005] Biocheck.Gent, Ghent University . 2023. Accessed 10 Feb 2023. https://biocheckgent.com/sites/default/files/2023-03/Pig_DE_V3.0.pdf.

[vms370041-bib-0006] Boyen, F. , F. Haesebrouck , A. Vanparys , et al. 2008. “Coated Fatty Acids Alter Virulence Properties of *Salmonella Typhimurium* and Decrease Intestinal Colonization of Pigs.” Veterinary Microbiology 132: 319–327. 10.1016/j.vetmic.2008.05.008. 18583068

[vms370041-bib-0007] Bundesanstalt für Landwirtschaft und Ernährung . 2018. Bericht Zur Markt—Und Versorgungslage Futtermittel 2018. Bonn, Germany: Bundesanstalt für Landwirtschaft und Ernährung.

[vms370041-bib-0008] Cappai, M. G. , C. Dimauro , M. Arlinghaus , S. J. Sander , W. Pinna , and J. Kamphues . 2020. “Subluminal Focal Lesions in Peyer's Patches in the Terminal Ileum of Pigs Fed With Different Physical Forms of One Same Diet.” Frontiers in Veterinary Science 7: 207. 10.3389/fvets.2020.00207. 32478102 PMC7242563

[vms370041-bib-0009] Chuppava, B. , V. Wilke , C. B. Hartung , et al. 2020. “Effect of a High Proportion of Rye in Compound Feed for Reduction of *Salmonella Typhimurium* in Experimentally Infected Young Pigs.” Microorganisms 8: 1629. 10.3390/microorganisms8111629. 33105623 PMC7690436

[vms370041-bib-0011] European Food Safety Authority (EFSA); European Centre for Disease Prevention and Control (ECDC) . 2023. “The European Union One Health 2022 Zoonoses Report.” EFSA Journal 21, no. 12: e8442. 10.2903/j.efsa.2023.8442. 38089471 PMC10714251

[vms370041-bib-0013] Fabà, L. , R. Litjens , J. Allaart , and P. R. van den Hil . 2020. “Feed Additive Blends Fed to Nursery Pigs Challenged With Salmonella.” Journal of Animal Science 98: skz382. 10.1093/jas/skz382. 31863091 PMC6978908

[vms370041-bib-0014] Food and Agriculture Organization of the United States (FAOSTAT) . Accessed July 2024. https://www.fao.org/faostat/en/#data/QCL.

[vms370041-bib-0016] Friend, D. W. , and T. M. Macintyre . 1970. “Effect of Rye Ergot on Growth and N‐Retention in Growing Pigs.” Canadian Journal of Comparative Medicine 34: 198–202. 4248440 PMC1319492

[vms370041-bib-0017] Gantois, I. , R. Ducatelle , F. Pasmans , et al. 2006. “Butyrate Specifically Down‐Regulates *Salmonella* Pathogenicity Island 1 Gene Expression.” Applied and Environmental Microbiology 72: 946–949. 10.1128/AEM.72.1.946-949.2006. 16391141 PMC1352287

[vms370041-bib-0018] Glitsø, L. , G. Brunsgaard , S. Højsgaard , B. Sandström , and K. B. Knudsen . 1998. “Intestinal Degradation in Pigs of Rye Dietary Fibre With Different Structural Characteristics.” British Journal of Nutrition 80: 457–468. 10.1017/S0007114598001536. 9924268

[vms370041-bib-0020] Hankel, J. , B. Chuppava , V. Wilke , et al. 2022. “High Dietary Intake of Rye Affects Porcine Gut Microbiota in a *Salmonella Typhimurium* Infection Study.” Plants 11: 2232. 10.3390/plants11172232. 36079614 PMC9460007

[vms370041-bib-0022] Hollmann, I. , J. B. Lingens , B. Chuppava , et al. 2022. “In Vitro Evaluation of Sodium Butyrate on the Growth of Three *Salmonella* Serovars Derived From Pigs at a Mild Acidic pH Value.” Frontiers in Veterinary Science 9: 937671. 10.3389/fvets.2022.937671. 35958300 PMC9360501

[vms370041-bib-0023] Jürgens, H. U. , G. Jansen , and C. B. Wegener . 2012. “Characterisation of Several Rye Cultivars With Respect to Arabinoxylans and Extract Viscosity.” Journal of Agricultural Science 4: 1–12. 10.5539/jas.v4n5p1.

[vms370041-bib-0024] Kamphues, J. , I. Brüning , S. Papenbrock , A. Möβeler , P. Wolf , and J. Verspohl . 2007. “Lower Grinding Intensity of Cereals for Dietetic Effects in Piglets?.” Livestock Science 109, no. 1–3: 132–134. 10.1016/j.livsci.2007.01.120.

[vms370041-bib-0025] Kump, F. W. S. , and U. Löhren . 2015. “Salmonellen Bekämpfung in der Primärproduktion–ein Kritischer Vergleich der Strategie und der Erfolge.” Veterinär Spiegel 25: 132–138.

[vms370041-bib-0026] Lawhon, S. D. , R. Maurer , M. Suyemoto , and C. Altier . 2002. “Intestinal Short‐Chain Fatty Acids Alter *Salmonella Typhimurium* Invasion Gene Expression and Virulence Through BarA/SirA.” Molecular Microbiology 46: 1451–1464. 10.1046/j.1365-2958.2002.03268.x. 12453229

[vms370041-bib-0029] McGhee, M. L. , and H. H. Stein . 2018. “Apparent and Standardized Ileal Digestibility of AA and Starch in Hybrid Rye, Barley, Wheat, and Corn Fed to Growing Pigs.” Journal of Animal Science 96, no. 8: 3319–3329. 10.1093/jas/sky206. 29939326 PMC6095345

[vms370041-bib-0030] McGhee, M. L. , and H. H. Stein . 2020. “The Apparent Ileal Digestibility and the Apparent Total Tract Digestibility of Carbohydrates and Energy in Hybrid Rye Are Different From Some Other Cereal Grains When Fed to Growing Pigs.” Journal of Animal Science 98, no. 7: skaa218. 10.1093/jas/skaa218. 32658254 PMC7394130

[vms370041-bib-0031] Miedaner, T. , and H. H. Geiger . 2015. “Biology, Genetics, and Management of Ergot (*Claviceps* spp.) in Rye, Sorghum, and Pearl Millet.” Toxins 7: 659–678. 10.3390/toxins7030659. 25723323 PMC4379517

[vms370041-bib-0033] Persson, K. , R. von Bothmer , M. Gullord , and E. Gunnarsson . 2006. “Phenotypic Variation and Relationships in Landraces and Improved Varieties of Rye (*Secale cereale* L.) From Northern Europe.” Genetic Resources and Crop Evolution 53: 857–866. 10.1007/s10722-004-6694-8.

[vms370041-bib-0027] QS Qualität und Sicherheit GmbH, Leitfaden QS Salmonellenmonitoring . 2023. https://www.q‐s.de/services/files/downloadcenter/h‐salmonellenmonitoring/2024/leitfaden/deutsch/Leitfaden_Salmonellenmonitoring_Schwein_01.01.2024.pdf.

[vms370041-bib-0034] Robert Koch‐Institut . 2019. “Infektionsepidemiologisches Jahrbuch Meldepflichtiger Krankheiten für 2018.” Last modified August, 2019. https://www.rki.de/DE/Content/Infekt/Jahrbuch/Jahrbuch_2018.pdf?__blob=publicationFile.

[vms370041-bib-0035] Robert Koch‐Institut . 2021. “Infektionsepidemiologisches Jahrbuch Meldepflichtiger Krankheiten für 2020.” Last modified March, 2021. https://www.rki.de/DE/Content/Infekt/Jahrbuch/Jahrbuch_2020.pdf?__blob=publicationFile.

[vms370041-bib-0036] Rodehutscord, M. , C. Rückert , H. P. Maurer , et al. 2016. “Variation in Chemical Composition and Physical Characteristics of Cereal Grains From Different Geno‐Types.” Archives of Animal Nutrition 70, no. 2: 87–107. 10.1080/1745039X.2015.1133111. 26829392

[vms370041-bib-0038] Sander, S. J. , J. Bullermann , M. Arlinghaus , J. Verspohl , and J. Kamphues . 2012. “The Influence of Grinding Intensity and Compaction of Diets on the Microbial Community in the Gastrointestinal Tract of Young Pigs.” Journal of Animal Science 90, no. S4: 16–18. 10.2527/jas.52522. 23365270

[vms370041-bib-0039] Schweine‐Salmonellen‐Verordnung . 2007. “Verordnung zur Verminderung der Salmonellenverbreitung durch Schlachtschweine (Schweine‐Salmonellen‐Verordnung) vom 13. März 2007.” Bundesgesetzblatt Jahrgang 10: 322–325.

[vms370041-bib-0041] Soliani, L. , G. Rugna , A. Prosperi , C. Chiapponi , and A. Luppi . 2023. “ *Salmonella* Infection in Pigs: Disease, Prevalence, and a Link Between Swine and Human Health.” Pathogens 12: 1267. 10.3390/pathogens12101267. 37887782 PMC10610219

[vms370041-bib-0048] Tan, FP. , E. Beltranena , and R. T. Zijlstra . 2021. “Resistant starch: Implications of dietary inclusion on gut health and growth in pigs: A review.” Journal of Animal Science and Biotechnology 12: 124. 34784962 10.1186/s40104-021-00644-5 PMC8597317

[vms370041-bib-0042] Van Immerseel, F. , J. Russell , M. Flythe , et al. 2006. “The Use of Organic Acids to Combat *Salmonella* in Poultry: A Mechanistic Explanation of the Efficacy.” Avian Pathology 35: 182–188. 16753609 10.1080/03079450600711045

[vms370041-bib-0044] Viana, C. , M. J. Sereno , L. D. S. Bersot , J. D. Kich , and L. A. Nero . 2020. “Comparison of Meat Juice Serology and Bacteriology for Surveillance of *Salmonella* in the Brazilian Pork Production Chain.” Food‐Borne Pathogens and Disease 17, no. 3: 194–201. 10.1089/fpd.2019.2712. 31661316

[vms370041-bib-0045] Visscher, C. , P. Winter , J. Verspohl , et al. 2009. “Effects of Feed Particle Size at Dietary Presence of Added Organic Acids on Caecal Parameters and the Prevalence of *Salmonella* in Fattening Pigs on Farm and at Slaughter.” Journal of Animal Physiology and Animal Nutrition 93: 423–430. 10.1111/j.1439-0396.2008.00821.x. 18537853

[vms370041-bib-0049] Visscher, C. , G. Klein , J. Verspohl , M. Beyerbach , J. Stratmann‐Selke , J. Kamphues , et al. 2011. “Serodiversity and Serological as Well as Cultural Distribution of Salmonella on Farms and in Abattoirs in Lower Saxony, Germany.” International Journal of Food Microbiology 146: 44–51. 21334757 10.1016/j.ijfoodmicro.2011.01.038

[vms370041-bib-0028] Vital, M. , A. Karch , and D. H. Pieper . 2017. “Colonic Butyrate‐Producing Communities in Humans: an Overview Using Omics Data.” mSystems 2, no. 6: e00130‐00117. 29238752 10.1128/mSystems.00130-17 PMC5715108

[vms370041-bib-0047] Wilke, V. 2020. “Effects of Increasing Rye or Rye and Rapeseed Meal (Extracted) Levels in Diets for Young Pigs Regarding Digestibility and Performance as Well as Diverse Properties (milieu/substrate) of the Digesta in the Alimentary Tract.” Ph.D. Thesis., University of Veterinary Medicine Hannover.

[vms370041-bib-0001] Viehzentrale Südwest GmbH . 2023. Accessed February 10, 2023. https://www.vz‐gmbh.de/media/2022/12/Leitfaden_Salmonellenmonitoring_Schwein_01.01.2023.pdf.

