# Supplementary material for: Examination of Salmonella Prevalence in Pigs Through Rye‐Based Feeding and Coarser Feed Structure Under Field Conditions
Source: Vet Med Sci. 2024 Sep 27;10(6):e70041. doi: 10.1002/vms3.70041 (PMC11430175; doi:10.1002/vms3.70041)
Supplement: Supplementary file 1 — Supporting Information [file VMS3-10-e70041-s001.docx]

Overview to the biosecurity on farms as to the Biosecurity Check Pig questionnaire, Ghent University, Belgium (Faculty of Veterinary Medicine)

| Parameter | Results |
| --- | --- |
| Purchase of breeding animals, piglets and semen | In none of the seven farms participating in the study were additional breeding animals (sows/gilts/boars) purchased during the study period. The situation was different for piglets. Farms 1, 2, 3, 4 and 7 were supplied with piglets 3 to 6 times a year. Farms 5 and 6 were even supplied with piglets more than 12 times per year. All farms paid attention to the required hygienic measures for the transport vehicles of the piglets. Farm 7 was the only farm that did not require proof that the purchased piglets came from a farm that had at least the same or higher hygiene status, as well as health management, as its own farm. |
| Animal transport, disposal of animal carcasses and manure | There was no transport of piglets from one farm to another on any of the farms. Likewise, drivers of the delivered piglets did not have access to the stables and always delivered to the farms via a separate loading ramp. The handling of carcasses was as follows: Only on farm 7, the bin was cleaned and disinfected regularly after emptying. None of the farms had a refrigerated carcass bin. After handling carcasses, either gloves were always worn or hands were always washed and disinfected after contact. |
| Supply of feed, water and equipment | Again, only Farm 7 differed from the other farms in this section. For example, only on Farm 7, there were specific hygiene measures that were applied to materials and special locks for tools used in the barn. Whereas on the six other farms no measures were taken. |
| Visitors and farmers | The hygiene measures for visitors and farmers were the same on all farms. Hands had to be washed and disinfected before entering the stables. Each visitor had his or her own footwear and the specified hygiene measures were carried out by both the farm manager and the staff. |
| Farm location | Each of the seven farms was located in a region with less than 300 pigs per km^2^. Farms 1-4 had another pig farm within 500m. Also on these farms, slurry from other farms was spread on fields 500m away. Regular transport of pigs belonging to other farms did not take place on any of the farms. Each farm was located in an area where wild boar populations occur. All farms described themselves as closed farms. |
| Interdepartmental measures, work procedures and equipment | Clothing and footwear was always changed between departments at Farm 7, sometimes at Farms 5 and 6 and never at Farms 1-4. The same applied to hand washing and disinfecting between departments. Disinfection tubs or boot washing facilities between the departments were available at farms 5-7. On farm 7, the younger animals were not treated first and then the older animals, as is the case on the other farms. Equipment for specific age groups was available at farms 5-7 and was only used for the respective groups. Each farm had specific instructions for cleaning and disinfecting the equipment. |
| Cleaning and disinfection | When it comes to cleaning and disinfection, all seven farms followed the given steps and cleaning times. All farms also cleaned and disinfected each compartment and barn after each production cycle, and allowed sufficient time for the barn to dry out and for the temperature to be adjusted. The driveways and aisles were also always cleaned and disinfected after each movement of pigs. A subsequent hygiene test after cleaning sometimes took place on farms 5 and 6. No tests were carried out at the other farms |
